# Author Correction: The role of charge in microdroplet redox chemistry

**DOI:** 10.1038/s41467-025-62749-z

**Published:** 2025-08-06

**Authors:** Joseph P. Heindel, R. Allen LaCour, Teresa Head-Gordon

**Affiliations:** 1 Kenneth S. Pitzer Theory Center and Department of Chemistry, Berkeley, CA USA; 2 Chemical Sciences Division, Lawrence Berkeley National Laboratory, Berkeley, CA USA; 3 Departments of Bioengineering and Chemical and Biomolecular Engineering University of CAlifornia, Berkeley, CA USA

**Keywords:** Catalysis, Surface chemistry, Molecular dynamics, Computational chemistry

Correction to: *Nature Communications* 10.1038/s41467-024-47879-0, published online 30 April 2024

The original version of this article did not cite ref. 20 (Colussi, A. J. Mechanism of hydrogen peroxide formation on sprayed water microdroplets. *J. Am. Chem. Soc*. **145**,16315-16317 (2023)), which is relevant for providing context to the work. The following sentence has been added at the end of the second paragraph of the Introduction: “Previous studies have suggested that HO-radicals, and consequently H_2_O_2_, can be formed at the surface because partially hydrated HO^−^ and H^+^ ions undergo electron transfer during encounters between charged microdroplets^20^, and/or because of the high electric field generated by contact electrification^21^.” Reference 21 (Lin, S., Cao, L. N. Y., Tang, Z. & Wang, Z. L. Size-dependent charge transfer between water microdroplets. *Proc. Natl Acad. Sci. USA* **120**, e2307977120 (2023)) was cited as ref. 31 in the original version. All the references following 20, 21 have been renumbered incrementing by two up to 31 and incrementing by one from 32 to 94.

