# Supplementary material for: The role of charge in microdroplet redox chemistry
Source: Nat Commun. 2024 Apr 30;15:3670. doi: 10.1038/s41467-024-47879-0 (PMC11519639; doi:10.1038/s41467-024-47879-0)
Supplement: Supplementary file 1 — Supplementary Information [file 41467_2024_47879_MOESM1_ESM.pdf]

Supplementary Information: The Role of Charge in Microdroplet Redox Chemistry

Joseph Heindel[1,2], R. Allen LaCour[1,2], Teresa Head-Gordon[1,2,3]

[1]Kenneth S. Pitzer Theory Center and Department of Chemistry
[2]Chemical Sciences Division, Lawrence Berkeley National Laboratory
[3]Departments of Bioengineering and Chemical and Biomolecular Engineering
University of California, Berkeley, CA, USA
corresponding author: thg@berkeley.edu

Supplementary Note 1: Calculation of Surface Tension The ReaxFF/CGeM model has been thoroughly validated for many properties of water[1] and the diffusion of H^+ and OH^-[2], the surface tension for this water model has not yet been reported. Our primary use of ReaxFF/CGeM in this work is to generate configurations for high-level electronic structure calculations, however, since we are dealing with interfacial systems, we would like to validate that the surface tension predicted by ReaxFF/CGeM is at least semi-quantitative. To this end, we have computed the surface tension of ReaxFF/CGeM using a slab geometry at 293K. We follow a similar protocol as that reported elsewhere.[3] Specifically, we compute the surface tension with three slab systems containing 512, 1024, and 2048 water molecules in boxes of length. All simulations were equilibrated for 1ns followed by 1ns of production simulation used for analysis. The reported uncertainties come from block averaging over five 200ps windows. All simulations use a time step of 0.25fs.

The surface tension can be computed from the pressure components in an NVT simulation,

$$\gamma = \frac{L_z}{2}\left[\langle P_{zz}\rangle - 0.5(\langle P_{xx}\rangle + \langle P_{yy}\rangle)\right] \tag{1}$$

In Eq. 1, γ is the surface tension, L_z is the box length in the z direction (which is normal to the interface), and $\langle P_{\alpha\alpha}\rangle$ is the diagonal component of the pressure tensor in the α direction ($\alpha = x, y, z$). The factor of 1/2 in Eq. 1 comes from the fact that there are two interfaces in a slab geometry.

Supplementary Note 2: Estimates of Ion Hydration Entropy In an attempt to understand how the hydration entropy of H^+ and OH^- change when there are excess ions in a droplet, we have computed the solvent accessible surface area (SASA) of all sampled clusters includng the 35 explicit solvent molecules using the Shrake-Rupley algorithm[4]. This is motivated by the observation that the SASA can be correlated to hydration entropy[5,6]. As seen in Fig. 1, there is virtually no change in SASA for OH^- and a very modest increase in SASA for H^+. This further justifies our focus on the change in hydration enthalpy in charged droplets over the change in hydration entropy.

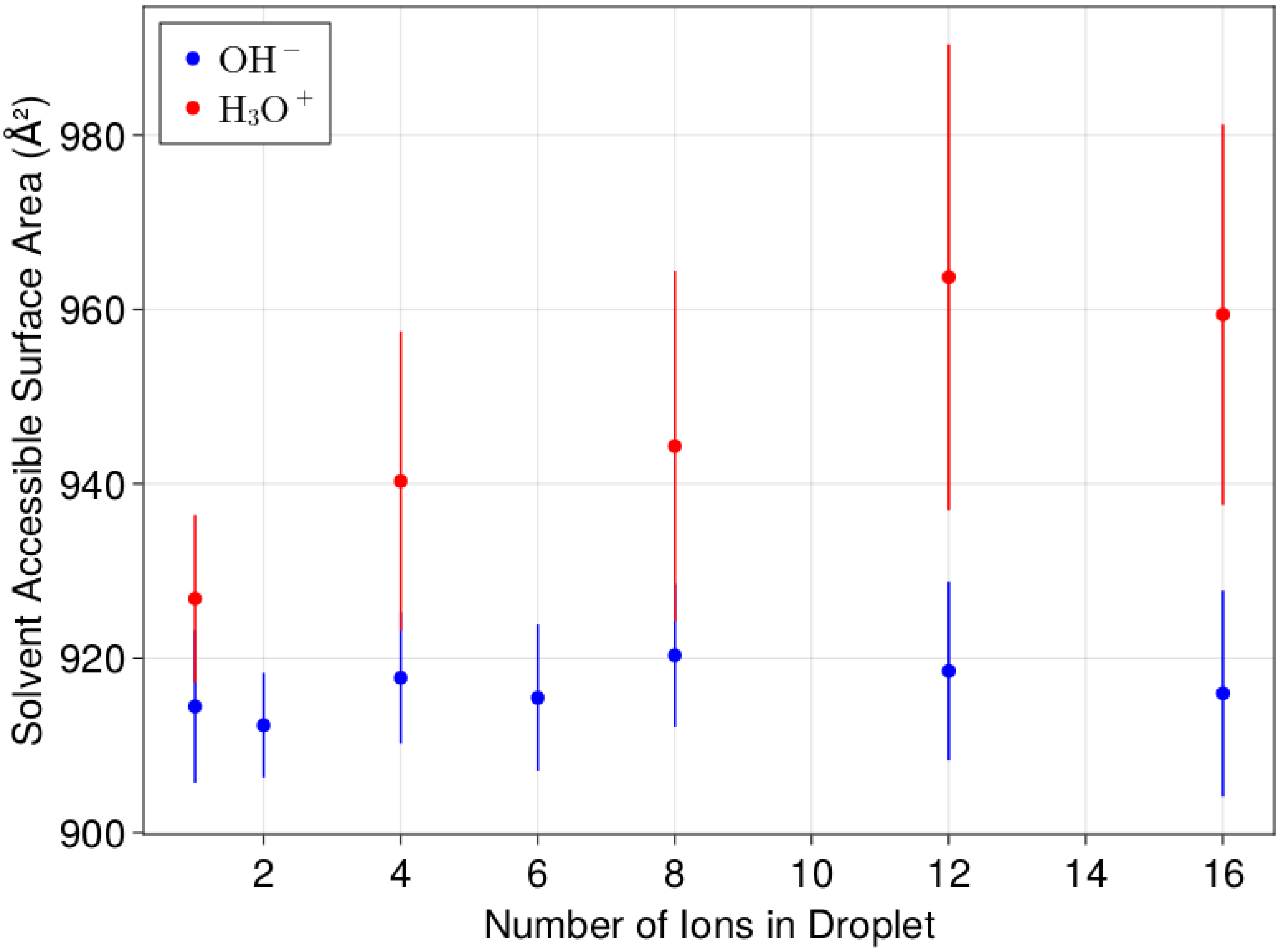

Supplementary Figure 1: *Solvent accessible surface area of $OH^-(H_2O)_{35}$ and $H_3O^+(H_2O)_{35}$ clusters.* We compute the solvent accessible surface area of $OH^-(H_2O)_{35}$ and $H_3O^+(H_2O)_{35}$ clusters as a measure of the importance of hydration entropy for the thermodynamics discussed in this paper. The small changes with increasing number of ions validates the expectation that the solvation free energy of an ion is dominated by the enthalpy. Error bars are standard deviations over 100 clusters.

Supplementary Note 3: Basis Set Superposition Error (BSSE) For systems of 35 monomers, as studied here, the BSSE correction would require spending nearly all of our computation time estimating the BSSE rather than computing the actual quantities of interest. Our approach to correcting for BSSE takes advantage of the fact that BSSE is nearly pairwise-additive and can be easily mapped onto the O-O distance[7]. Our approach is to fit the dependence of BSSE to an exponential functional form, $E_{BSSE} = a\exp(-bR_{OO})$ based on a relaxed scan of the $(H_2O)_2$, $OH^-(H_2O)$, and $H_3O^+(H_2O)$ potential energy surfaces. The corresponding BSSE correction is then computed as a sum over all dimers applying the appropriate fitted parameters for that dimer. The curves and fit parameters from the dimer scans are shown in Fig. 2.

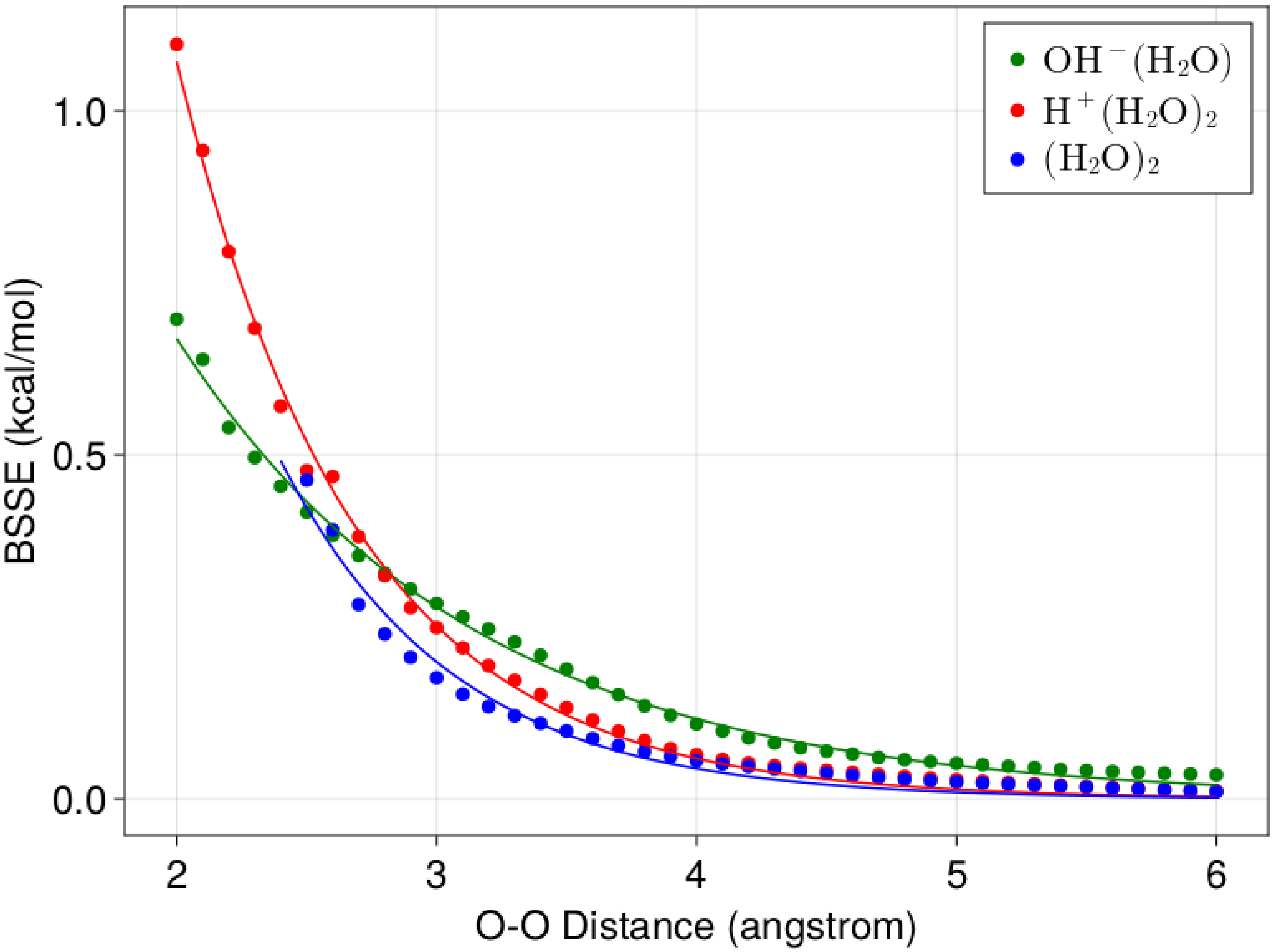

Supplementary Figure 2: *Basis set superposition error (BSSE) computed at the ωB97M-V/aug-cc-pVDZ level of theory.* The BSSE is computed for $(H_2O)_2$, $OH^-(H_2O)$, and $H_3O^+(H_2O)$ as a function of oxygen-oyxgen distance. These scans are then fit to an exponential. This approach is known to be capable of accurately reproducing the full BSSE.[7] The parameters of the exponential fit are $a = 18.273$, $b = 1.506$ for $(H_2O)_2$, $a = 3.848$, $b = 0.8746$ for $OH^-(H_2O)$, and $a = 19.462$, $b = 1.449$ for $H_3O^+(H_2O)$.

Supplementary References

[1] Itai Leven, Hongxia Hao, Akshaya Kumar Das, and Teresa Head-Gordon. A reactive force field with coarse-grained electrons for liquid water. *J. Phys. Chem. Lett.*, 11(21):9240–9247, 2020.

[2] Itai Leven, Hongxia Hao, Songchen Tan, Xingyi Guan, Katheryn A Penrod, Dooman Akbarian, Benjamin Evangelisti, Md Jamil Hossain, Md Mahbubul Islam, Jason P Koski, et al. Recent advances for improving the accuracy, transferability, and efficiency of reactive force fields. *J. Chem. Theo. Comp.*, 17(6):3237–3251, 2021.

[3] Maria Carolina Muniz, Thomas E Gartner, Marc Riera, Christopher Knight, Shuwen Yue, Francesco Paesani, and Athanassios Z Panagiotopoulos. Vapor–liquid equilibrium of water with the mb-pol many-body potential. *J. Chem. Phys.*, 154(21), 2021.

[4] Andrew Shrake and John A Rupley. Environment and exposure to solvent of protein atoms. lysozyme and insulin. *JJ. Mol. Bio.*, 79(2):351–371, 1973.

[5] Emilio Gallicchio, MM Kubo, and Ronald M Levy. Enthalpy- entropy and cavity decomposition of alkane hydration free energies: numerical results and implications for theories of hydrophobic solvation. *J. Phys. Chem. B*, 104(26):6271–6285, 2000.

[6] Junmei Wang and Tingjun Hou. Develop and test a solvent accessible surface area-based model in conformational calculations. *J. Chem. Inform. Model.*, 52(5):1199–1212, 2012.

[7] Joseph P Heindel and Sotiris S Xantheas. The many-body expansion for aqueous systems revisited: I. water–water interactions. *J. Chem. Theo. Comp.*, 16(11):6843–6855, 2020.
